# A brief report on the association of preoperative hematological indices and acute deep vein thrombosis following total hip arthroplasty for osteoarthritis

**DOI:** 10.2478/jccm-2025-0018

**Published:** 2025-04-30

**Authors:** Razvan Marian Melinte, Matei Florin Negrut, Daniel Oltean-Dan, Adrian Dumitru Ivanescu, Tudor-Mihai Magdas, Oana Antal, Adela Hilda Onutu, Marian Andrei Melinte, Robert Bolcas

**Affiliations:** 1st Department of Orthopedics and Traumatology, “Iuliu Hațieganu” University of Medicine and Pharmacy, Cluj-Napoca, Romania; Department of Orthopedics, Regina Maria Health Network, Targu Mures, Romania; “Iuliu Hațieganu” University of Medicine and Pharmacy, Cluj-Napoca, Romania; Department of Orthopedics and Traumatology, County Emergency Hospital, Cluj-Napoca, Romania; MedLife Humanitas Hospital, Cluj-Napoca, Romania; Department of Anatomy, George Emil Palade University of Medicine, Pharmacy, Science, and Technology of Targu Mures, Targu Mures, Romania; Department of Anatomy, “Iuliu Hațieganu” University of Medicine and Pharmacy, Cluj-Napoca, Romania; 2nd Department of Anesthesia and Intensive Care, “Iuliu Hațieganu” University of Medicine and Pharmacy, Cluj-Napoca, Romania; Department of Anesthesia and Intensive Care, Orthopaedics and Traumatology Clinic, County Emergency Hospital, Cluj-Napoca, Romania; George Emil Palade University of Medicine, Pharmacy, Science, and Technology of Targu Mures, Romania

**Keywords:** total hip arthroplasty, deep vein thrombosis, monocyte-to-lymphocyte ratio, neutrophil-to-lymphocyte ratio, platelets-to-lymphocyte ratio, systemic inflammatory index

## Abstract

**Introduction:**

Total hip arthroplasty (THA) is a standard orthopedic procedure. Deep vein thrombosis (DVT) and pulmonary embolism are potential life-threatening postoperative complications.

**Aim of the study:**

This study aimed to assess the prognostic value of systemic inflammatory indices [monocyte-to-lymphocyte ratio (MLR), neutrophil-to-lymphocyte ratio (NLR), platelets-to-lymphocyte ratio (PLR), systemic inflammatory index (SII), systemic inflammation response index (SIRI), and aggregate index of systemic inflammation (AISI)] and their potential association with acute postoperative DVT.

**Materials and methods:**

We designed a multicentric, retrospective, observational cohort study, including adult patients undergoing elective HTA. Patients were divided into two groups, the DVT and non-DVT groups. We investigated the development of acute DVT early, and at 4 weeks after surgery and also registered length of hospital stay and mortality. All demographic data and laboratory data, hematological indices were extracted from patients files.

**Results:**

199 patients were included. Of those, 12 (6.03%) developed DVT and 187 (93.97%) did not. There was no statistically significant difference between patient age, gender, BMI, smoking status or comorbidities. No difference was founds between the two groups regarding median values of MLR (0.31 vs 0.27, p=0.12), NLR (3.16 vs 2.42, p=0.27), PLR (163.39 vs 123.01, p=0.27), SII (660.26 vs 568.52, p=0.33), SIRI (67.5 vs 65.26, p=0.89) and AISI (302.35 vs 290.48, p=0.85). Length of hospital stay was not significantly different (median 9 days in the DVT group vs 7 days in the non-DVT group, p=0.38), but mortality was significantly higher in the DVT group (3 deaths vs none in the non-DVT group, p<0.001).

**Conclusion:**

MLR, NLR, PRL, SII, SIRI and AISI were not associated with the development of acute DVT following HTA in our study population.

## Introduction

Total hip arthroplasty (THA), often referred to as „the operation of the century,” is a standard orthopedic procedure for both young and elderly people seeking to regain an active lifestyle and improve overall quality of life, without pain [1,2].

As the main indication of this specific therapy is hip osteoarthritis in the elderly, a growing population with an increased age and likelihood of associated disease, the perioperative complication rates are rapidly rising [3]. Deep vein thrombosis (DVT) and pulmonary embolism (PE) are being acknowledged as frequent postoperative complications, increasing length of hospital stay, mortality, and costs [1,4,5].

With reported incidences of thrombus formation in up to 60% of arthroplasty cases without prophylaxis [6,7], anticoagulants have become standard practice, as recommended by several guidelines, including the ERAS and ESAIC guidelines, where the antithrombotic prophylaxis represents a strong recommendation [8,9]. There are independent risk factors for developing post-procedural thrombosis, including non-modifiable factors like age and female gender, elevated D-dimer values, and the type of hip arthroplasty procedure, such as revision THA [10,11,12,13,14].

Recent research has explored more individual-specific predictive factors for thrombosis development, such as inflammatory markers like CRP and IL-6, alongside various hematological ratios [15,16,17,18]. This study aims to evaluate the association between hematological ratios (indices) — monocyte-to-lymphocyte ratio (MLR), neutrophil-to-lymphocyte ratio (NLR), platelets-to-lymphocyte ratio (PLR), systemic inflammatory index (SII), systemic inflammation response index (SIRI), and aggregate index of systemic inflammation (AISI) — and acute thrombosis following THA surgery. We investigated the development of DVT in the first four weeks post-surgery, as well as length of hospital stay and mortality.

## Materials and methods

### Patient selection

We conducted a multicentric, retrospective, observational cohort study, including consecutive patients undergoing elective total hip arthroplasty for osteoarthritis, admitted to the Departments of Orthopedics of the Regina Maria Health Network, Targu Mures, Humanitas MedLife Hospital, Cluj-Napoca, and the Emergency County Hospital Cluj-Napoca, Romania, from June 2021 to December 2023. Exclusion criteria were age below 18 years old, history of previous DVT and pulmonary embolism, and postoperative blood transfusion requirements.

The study was conducted in accordance with the Declaration of Helsinki and approved by the Ethics Committees of Regina Maria Health Network, Targu Mures (Approval No: 84 Date: 11.05.2021), Humanitas MedLife Hospital, Cluj-Napoca (Approval No: 1 Date: 28.01.2019) and Emergency County Hospital Cluj-Napoca, Romania (Approval No: 1123 Date: 14.05.2021). Written informed consent was obtained from the patient who agreed to take part in the study.

### Data collection

Demographic and medical data were collected from the hospital’s computerized database and patients’ charts, including age, gender, smoking status, presence of systemic hypertension (SHT), chronic coronary disease (CCD), atrial fibrillation (AFib), congestive heart failure (CHF), dyslipidemia, type II diabetes mellitus (T2DM), chronic kidney disease (CKD), chronic venous insufficiency (CVI), obesity, or known malignancy.

Laboratory parameters were collected preoperatively in the morning before surgery. They included hemoglobin concentration, hematocrit, neutrophil, lymphocyte, monocyte, and thrombocyte counts, activated partial thromboplastin time (APTT), international normalized ratio (INR), serum glucose concentration, serum urea, and creatinine levels. Systemic inflammation indices were calculated using the following formulas:

– MLR = total number of monocytes/total number of lymphocytes
– NLR = total number of neutrophils/total number of lymphocytes
– PLR = total number of platelets/total number of lymphocytes
– SII = (total number of neutrophils × total number of platelets)/total number of lymphocytes
– SIRI = (total number of monocytes × total number of platelets)/total number of lymphocytes
– AISI = (total number of neutrophils × total number of monocytes × total number of platelets)/total number of lymphocytes

### Surgical technique

Surgery was carried out by two experienced surgical teams, using the same technique in all patients. Via an anterolateral approach, a Zimmer trilogy, Harris-Galante (Zimmer, IN, USA) prosthesis with an uncemented Metabloc Stem System was implanted, using either a metal or ceramic head. Thromboprophylaxis was ensured using subcutaneous enoxaparin, starting 12 hours preoperatively and up to a month following surgery, and appropriate anti-embolism stockings.

The follow-up for DVT was performed using Doppler ultrasonography at discharge and after four weeks.

### Statistical analysis

The patients were divided into two groups, the DVT and non-DVT groups. Statistical analysis was performed using SPSS Statistics v29.0.1 (IBM Corp., Armonk, NY, USA). Continuous variables were reported as mean ± standard deviation (SD) and compared using Student’s t-test if normally distributed. Variables not normally distributed were reported using median and interquartile range (Q1-Q3) and compared using Mann-Whitney U. Normality was tested using Shapiro-Wilk, and equality of variances using Levene’s test. Categorical variables were reported as frequencies and compared using either the Chi-Square test, Chi-Square with Yates correction, or Fisher’s exact test. A two-tailed p<0.05 was considered statistically significant.

## Results

Of the 208 patients initially screened for inclusion, 4 were excluded due to a history of previous DVT, and 5 were excluded due to postoperative transfusion requirements. One hundred ninety-nine patients were included in the final analysis: 12 (6.03%) in the DVT group and 187 (93.97%) in the non-DVT group.

A summary of demographic and medical data is provided in Table 1. No statistically significant differences existed between patient age, gender and BMI. The frequency of comorbidities - such as hypertension (HT), chronic coronary disease (CCD), atrial fibrillation (AFib), congestive heart failure (CHF), dyslipidemia, type II diabetes mellitus (T2D), chronic kidney disease (CKD), chronic venous insufficiency (CVI), obesity, known malignancy or smoking status - was not significantly different between the DVT and non-DVT groups. Preoperative hematological parameters, systemic inflammation indices and serum parameters are shown in Table 2. No statistically significant differences were found between patient’s preoperative hemoglobin and hematocrit, or in the neutrophil, lymphocyte, monocyte or thrombocyte counts. Systemic inflammation indices did not differ significantly between the two groups. Blood glucose, APTT, INT, serum urea, and creatinine were non significantly different.

**Table 1.** Patient comorbidities and demographic data

	**All patients (n=199)**	**DVT (n=12)**	**non-DVT (n=187)**	**p**
Age (years) (mean±SD)	65.56±10.81	70.42±9.99	65.25±10.81	0.13
Gender				0.18
Male, n (%)	104 (52.26)	4 (3.85)	100 (96.15)	
Female, n (%)	95 (47.74)	8 (8.42)	87 (91.58)	
BMI (kg/m^2^)	27.35 ± 4.6	28.08±3.92	27.31±4.65	0.54
SHT, n (%)	147 (73.87)	8 (66.66)	139 (74.33)	0.81
CCD, n (%)	76 (38.19)	4 (33.33)	72 (38.50)	0.86
AFib, n (%)	9 (4.52)	1 (8.33)	8 (4.27)	0.99
Smoker, n (%)	6 (3.02)	1 (8.33)	5 (2.67)	0.76
CHF, n (%)	11 (5.52)	0 (0)	11 (5.88)	0.99
Dyslipidemia, n (%)	8 (4.02)	1 (8.33)	6 (3.21)	0.35
T2DM, n (%)	21 (10.55)	2 (16.66)	19 (10.16)	0.74
Obesity, n (%)	11 (5.53)	3 (25)	8 (4.27)	0.54
Malignancy, n (%)	8 (4.02)	0 (0)	8 (4.27)	0.99
CKD, n (%)	2 (1)	0 (0)	2 (1.07)	0.99
Varicose veins, n (%)	14 (7.04)	1 (8.33)	13 (6.95)	0.79

BMI, body mass index; SHT, systemic hypertension; CCD, chronic coronary disease; AFib, atrial fibrillation; CHF, congestive heart failure; T2DM, type 2 diabetes mellitus; CKD, chronic kidney disease.

**Table 2.** Laboratory parameters of the patients

**Parameter median (Q1-Q3)**	**All patients (n=199)**	**DVT (n=12)**	**non-DVT (n=187)**	**p**
Hb (g/dL)	13.8 (12.83–14.77)	13.71 (12.53 – 14.45)	13.8 (12.8 – 14.8)	0.42
Hct (%)	41.7 (39.13–44.4)	41.06 (37.23–42.26)	41.8 (39.25 – 44.42)	0.28
Neu (×10^3^/µL)	4.84 (3.7–5.98)	4.47 (4.12–6.26)	4.85 (3.69–5.98)	0.66
Ly (×10^3^/µL)	1.97 (1.51–2.55)	1.56 (1.09–2.44)	1.98 (1.55–2.55)	0.18
Mo (×10^3^/µL)	0.51 (0.41–0.68)	0.47 (0.3–0.64)	0.51 (0.41–0.68)	0.60
Tr (×10^3^/µL)	246 (207.5–291.4)	210.1 (178.5–284.25)	246.4 (211.55–291.4)	0.23
MLR	0.27 (0.2–0.37)	0.36 (0.22–0.53)	0.27 (0.2–0.36)	0.12
NLR	2.45 (1.79–3.19)	3.16 (1.7–5.52)	2.42 (1.81–3.13)	0.27
PLR	124.05 (96.96–164.48)	163.39 (114.32–175.73)	123.01 (96.96–162.51)	0.27
SII	572.52 (429.22–846.22)	660.26 (489.95–1100.5)	568.52 (429.22–823.18)	0.33
SIRI	65.26 (49.05 – 85.63)	67.5 (40.62–94.85)	65.26 (49.73–85.31)	0.89
AISI	290.48 (199.83–480.87)	302.35 (182.43–761.33)	290.48 (204.22–477.56)	0.85
Glycemia (mg/dL)	102 (92–115)	106.49 (83.5–115.75)	102 (92–115)	0.81
APTT (seconds)	25.85 (23.9–28.13)	27.45 (27.05–31)	25.8 (23.9–28.1)	0.09
INR	0.98 (0.93–1.08)	1 (0.96–1.06)	0.98 (0.93–1.08)	0.61
Serum urea (mg/dL)	34.8 (28.35–42.9)	29 (21.88–38.13)	35.3 (28.8–42.9)	0.13
Serum Creatinine (mg/dL)	0.8 (0.72–0.94)	0.93 (0.62–1.03)	0.8 (0.73–0.94)	0.60
LOS (days)	7 (6–10)	9 (7.75–11.25)	7 (6–10)	0.38
Death (number)	3	3	0	<0.001

Hb, haemoglobin; Hct, haematocrit; Neu, neutrophil count; Ly, lymphocyte count; Mo, monocyte count; Tr, thrombocyte counts; MLR, monocyte-to-lymphocyte ratio; NLR, neutrophil-to-lymphocyte ratio; PLR, platelets-to-lymphocyte ratio; SII, systemic inflammatory index; SIRI, systemic inflammation response index (SIRI); AISI, aggregate index of systemic inflammation; APTT, activated partial thromboplastin time; INR, international normalised ratio; LOS, length of hospital stay;

We found an elevated MLR median (Q1-Q3)=0.36 (0.22–0.53), NLR=3.16 (1.7–5.52), PLR=163.39 (114.32–175.73), SII=660.26 (489.95–1100.5), SIRI=67.5 (40.62–94.85) and AISI=302.35 (182.43–761.33) in DVT group, but still with no statistical significance.

Length of hospital stay was not statistically different between the two groups However, three patients died in the DVT group, while none died in the non-DTV group (p<0.001).

## Discussion

Hematological ratios are being explored as novel markers of inflammation, closely associated with various pathologies, ranging from acute inflammatory conditions to chronic diseases that predispose to a prothrombotic state [19].

The Platelet-to-Lymphocyte Ratio (PLR) is recognized as an indicator of systemic response in acute inflammatory pathologies. It reflects to some extent, the severity of systemic inflammation, cytokine storm, and subsequent prothrombotic environment [19,20].

Recent studies investigate the association between these ratios and the risk of deep vein thrombosis due to their ease of calculation and low cost [19,20,21,22].

Our results show no statistically significant differences in hematological indices between the DVT and non-DVT groups, probably because of the small population involved. As opposed to our results, it has been reported that higher preoperative and postoperative Neutrophil-to-Lymphocyte Ratio (NLR) and lower postoperative PLR are significantly associated with DVT. However, they cannot accurately predict DVT associated with orthopedic surgery [22].

In a previous study conducted by our team, higher MLR, NLR, PLR, SII, SIRI, and AISI were all associated with an increased risk of DVT in total knee arthroplasty (TKA) patients [16]. As TKA is known to be more thrombogenic than THA [23], these inflammatory biomarkers might be more useful in high-risk patients. Moreover, the low incidence of DVT in our current study population might lead to a lack of statistical power required to rule out a significant difference in preoperative hematological ratios between the DVT and non-DVT groups.

A meta-analysis revealed modest sensitivity (Se) and specificity (Sp) for the Monocyte-to-Lymphocyte Ratio (MLR), with Se ranging from 0.54 to 0.81 and Sp from 0.78 to 0.81. For NLR, Se was 0.72, and Sp was 0.74, and for PLR, Se was 0.77, and Sp was 0.75. These values individually lack sufficient predictive power for thrombosis risk assessment, rendering them unsuitable as standalone screening tools [24].

However, since most patients undergo preoperative hemogram evaluation and these ratios can be easily calculated at no additional cost, future studies may report these values. They could serve as components of a predictive score, in combination with other parameters and risk factors, to enhance predictive power. Therefore, while individual values may not be relevant, their integration into specific scoring systems may confer predictive value. Achieving this requires a large sample size to determine if there is a significant correlation, which was not attained in this study but has been reported in the literature [16,22,25]. A future prospective study might give new answers regarding the value of these hematological indices in predictiong postoperative deep thrombosis after joint arthroplasty.

Our study has several limitations, including a small sample size and a retrospective design. Doppler ultrasonography was only performed at discharge and four weeks post-hospital discharge, limited to the operated limb, which may have missed thrombotic events in the non-operated limb. Additionally, preoperative ultrasound data of the limbs were not available, a significant limitation considering recent findings indicating a prevalence of preoperative DVT in non-fracture patients awaiting total hip arthroplasty [11].

## Conclusions

Preoperative values of MLR, NLR, PLR, SII, SIRI, and AISI were not associated with an increased risk of DVT in the studied THA patients. However, these ratios might prove useful in very high-risk patients, and further studies, focusing on different patient subpopulations are warranted.

## References

[1] Learmonth ID, Young C, Rorabeck C (2007). The operation of the century: total hip replacement. Lancet.

[2] Ferguson RJ, Palmer AJ, Taylor A, Porter ML, Malchau H, Glyn-Jones S (2018). Hip replacement. Lancet.

[3] Murphy BPD, Dowsey MM, Choong PFM (2018). The Impact of Advanced Age on the Outcomes of Primary Total Hip and Knee Arthroplasty for Osteoarthritis: A Systematic Review. JBJS Rev.

[4] Zhang ZH, Shen B, Yang J, Zhou ZK, Kang PD, Pei FX (2015). Risk factors for venous thromboembolism of total hip arthroplasty and total knee arthroplasty: a systematic review of evidences in ten years. BMC Musculoskelet Disord.

[5] Zhang J, Chen Z, Zheng J, Breusch SJ, Tian J (2015). Risk factors for venous thromboembolism after total hip and total knee arthroplasty: a meta-analysis. Arch Orthop Trauma Surg.

[6] Falck-Ytter Y, Francis CW, Johanson NA (2012). Prevention of VTE in orthopedic surgery patients: Antithrombotic Therapy and Prevention of Thrombosis, 9th ed: American College of Chest Physicians Evidence-Based Clinical Practice Guidelines. Chest.

[7] Piovella F, Wang CJ, Lu H (2005). Deep-vein thrombosis rates after major orthopedic surgery in Asia. An epidemiological study based on postoperative screening with centrally adjudicated bilateral venography. J Thromb Haemost.

[8] Wainwright TW, Gill M, McDonald DA (2020). Consensus statement for perioperative care in total hip replacement and total knee replacement surgery: Enhanced Recovery After Surgery (ERAS^®^) Society recommendations. Acta Orthop.

[9] Afshari A, Ageno W, Ahmed A, ESA VTE Guidelines Task Force (2018). European Guidelines on perioperative venous thromboembolism prophylaxis: Executive summary. Eur J Anaesthesiol.

[10] Frassanito L, Vergari A, Nestorini R (2020). Enhanced recovery after surgery (ERAS) in hip and knee replacement surgery: description of a multidisciplinary program to improve management of the patients undergoing major orthopedic surgery. Musculoskelet Surg.

[11] Yao Y, Chai S, Qiao L, Jiang Q, Xu R (2024). An analysis of the prevalence and risk factors of deep vein thrombosis in non-fracture patients awaiting total hip arthroplasty: a retrospective study of 1244 cases. J Orthop Surg Res.

[12] Wakabayashi H, Hasegawa M, Niimi R, Sudo A (2015). Clinical analysis of preoperative deep vein thrombosis risk factors in patients undergoing total hip arthroplasty. Thromb Res.

[13] Kawai T, Goto K, Kuroda Y, Matsuda S (2020). Lower Activity and Function Scores Are Associated with a Higher Risk of Preoperative Deep Venous Thrombosis in Patients Undergoing Total Hip Arthroplasty. J Clin Med.

[14] Imai N, Miyasaka D, Shimada H, Suda K, Ito T, Endo N (2017). Usefulness of a novel method for the screening of deep vein thrombosis by using a combined D-dimer- and age-based index before total hip arthroplasty. PLoS One.

[15] Zacho J, Tybjaerg-Hansen A, Nordestgaard BG (2010). C-reactive protein and risk of venous thromboembolism in the general population. Arterioscler Thromb Vasc Biol.

[16] Melinte RM, Arbănași EM, Blesneac A (2022). Inflammatory Biomarkers as Prognostic Factors of Acute Deep Vein Thrombosis Following the Total Knee Arthroplasty. Medicina (Kaunas).

[17] Christiansen SC, Naess IA, Cannegieter SC, Hammerstrøm J, Rosendaal FR, Reitsma PH (2006). Inflammatory cytokines as risk factors for a first venous thrombosis: a prospective population-based study. PLoS Med.

[18] Roumen-Klappe EM, den Heijer M, van Uum SH, van der Ven-Jongekrijg J, van der Graaf F, Wollersheim H (2002). Inflammatory response in the acute phase of deep vein thrombosis. J Vasc Surg.

[19] Qu R, Ling Y, Zhang YH (2020). Platelet-to-lymphocyte ratio is associated with prognosis in patients with coronavirus disease-19. J Med Virol.

[20] Gasparyan AY, Ayvazyan L, Mukanova U, Yessirkepov M, Kitas GD (2019). The Platelet-to-Lymphocyte Ratio as an Inflammatory Marker in Rheumatic Diseases. Ann Lab Med.

[21] Akcal MA, Eke I (2021). Post-Operative Red Cell Distribution Width Increase May Predict Mortality in Patients Operated for Hip Fracture. Clin Lab.

[22] Yao C, Zhang Z, Yao Y, Xu X, Jiang Q, Shi D (2018). Predictive value of neutrophil to lymphocyte ratio and platelet to lymphocyte ratio for acute deep vein thrombosis after total joint arthroplasty: a retrospective study. J Orthop Surg Res.

[23] Gionis MN, Ioannou CV, Katsamouris AN (2013). The study of the thrombin generation mechanism and the effect of low molecular weight heparin as thromboprophylaxis in patients undergoing total knee and hip replacement. Thromb Res.

[24] Festa E, Ascione T, Bernasconi A (2022). Diagnostic Performance of Neutrophil to Lymphocyte Ratio, Monocyte to Lymphocyte Ratio, Platelet to Lymphocyte Ratio, and Platelet to Mean Platelet Volume Ratio in Periprosthetic Hip and Knee Infections: A Systematic Review and Meta-Analysis. Diagnostics (Basel).

[25] Karadeniz S, Yurtbay A (2022). Predicting mortality rate in elderly patients operated for hip fracture using red blood cell distribution width, neutrophil-to-lymphocyte ratio, and Nottingham Hip Fracture Score. Jt Dis Relat Surg.

